# Potential of MXenes in Water Desalination: Current Status and Perspectives

**DOI:** 10.1007/s40820-020-0411-9

**Published:** 2020-03-12

**Authors:** Ihsanullah Ihsanullah

**Affiliations:** Center for Environment and Water, Research Institute, King Fahd University of Petroleum and Minerals, Dhahran, 31261 Saudi Arabia

**Keywords:** MXenes, 2D materials, Metal carbides, Capacitive deionization, Solar desalination, Water desalination

## Abstract

MXenes, novel 2D transition metal carbides, have emerged as wonderful nanomaterials and a superlative contestant for a host of applications. The tremendous characteristics of MXenes, i.e., high surface area, high metallic conductivity, ease of functionalization, biocompatibility, activated metallic hydroxide sites, and hydrophilicity, make them the best aspirant for applications in energy storage, catalysis, sensors, electronics, and environmental remediation. Due to their exceptional physicochemical properties and multifarious chemical compositions, MXenes have gained considerable attention for applications in water treatment and desalination in recent times. It is vital to understand the current status of MXene applications in desalination in order to define the roadmap for the development of MXene-based materials and endorse their practical applications in the future. This paper critically reviews the recent advancement in the synthesis of MXenes and MXene-based composites for applications in desalination. The desalination potential of MXenes is portrayed in detail with a focus on ion-sieving membranes, capacitive deionization, and solar desalination. The ion removal mechanism and regeneration ability of MXenes are also summarized to get insight into the process. The key challenges and issues associated with the synthesis and applications of MXenes and MXene-based composites in desalination are highlighted. Lastly, research directions are provided to guarantee the synthesis and applications of MXenes in a more effective way. This review may provide an insight into the applications of MXenes for water desalination in the future.

## Highlights

- A broad overview of MXenes and MXene-based nanomaterials in desalination is presented.
- Recent advancement in the synthesis of MXenes for applications in desalination is critically evaluated. Salt removal mechanisms and regeneration capability of MXenes are appraised.
- Current challenges and future prospect of MXenes in desalination are highlighted. Research directions are provided to safeguard the applications of MXenes in future desalination.

## Graphical abstract

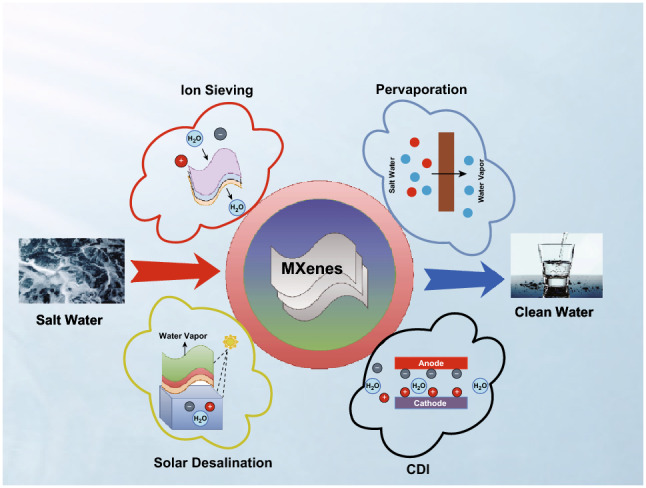

## Introduction

**Fig. 1:**
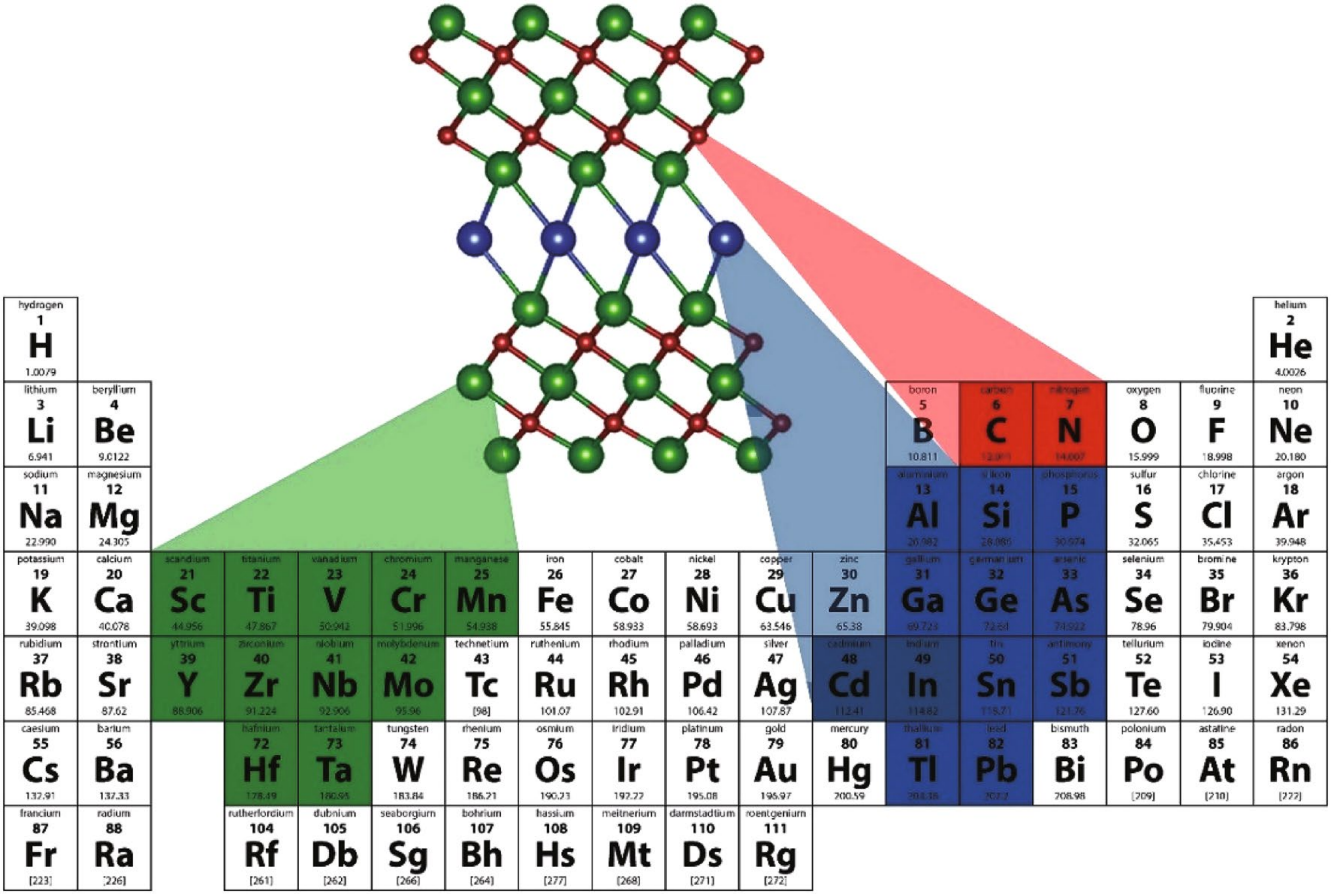
Elements in the periodic table that are known to form M_*n* + 1_AX_*n*_ phases. Reprinted with the permission from Ref. [17]. Copyright © (2019) Elsevier Ltd and Techna Group S.r.l

MXenes (pronounced “maxines”), a new family of 2D transition metal carbides, nitrides, and carbonitrides, were discovered by researchers at Drexel University in 2011 [1–4]. The first-ever MXene comprised of 2D titanium carbide (Ti_3_C_2_) was synthesized by selectively etching the “A” (Al atoms) in layered hexagonal ternary carbide, Ti_3_AlC_2_, with hydrofluoric acid (HF) at room temperature [1]. MXenes are represented by the general formula M_*n*+1_X_*n*_T_*x*_ (*n *= 1–3) and are derived from the precursor MAX phase (M_*n*+1_AX_*n*_), where M is an early transition metal, X is carbon and/or nitrogen, A represents an element from groups 12 to 16, T denotes the surface termination groups such as fluorine (−F), oxygen (=O), chlorine (−Cl), and hydroxyl (–OH), and *x* represents the number of surface functionalities [2, 5–13]. In the MAX phase, A layer is sandwiched within octahedral M_*n*+1_X_*n*_, with strong M–X bond and relatively weak M–A bond [14–16]. MAX phases (M_*n*+1_AX_*n*_) forming elements of the periodic table are highlighted in Fig. 1 [17]. The interlayer spacing of MXenes is usually in the range of 1 nm and is dependent on the value of *n* in MXenes (M_*n*+1_X_*n*_T_*x*_) [13].

So far, nearby 30 MXenes compositions are reported in the literature that have been synthesized from the MAX phase precursors primarily by incorporating two or more transition metals in the M layers [5, 18–21]. Among them, titanium-based MXenes (such as Ti_3_C_2_T_*x*_ and Ti_2_CT_*x*_) are extensively employed in environmental applications [1, 18]. Besides carbides, other MXenes structures with nitride and carbonitride have also been employed in various fields [19, 22–25]. Owing to their unique layered structures and 2D morphology, MXenes can be easily exploited to form a composite with other materials to enhance their properties [25–30].

MXenes have arisen a significant consideration of scientists and academicians due to their fascinating chemical, mechanical, electronic, and magnetic properties [18]. The incredible features of MXenes including, but not limited to, high surface area, environmentally friendly nature, biocompatibility, activated metallic hydroxide sites, ease of functionalization, antibacterial properties, high metallic conductivity, and hydrophilicity make them an ideal candidate for various applications such as environmental remediation, energy storage, electronics, sensors, and catalysis [6, 14, 16, 28, 31, 32]. The exceptional electrical conductivity (~ 6500 S m^−1^) and high surface area of MXenes (up to 347 m^2^ g^−1^) are essential characteristics for their applications in conductive films, electrical measurements, adsorption, and energy storage [6, 13, 33]. Recent studies demonstrated that MXenese also holds a prominent future in water splitting applications [34, 35]. MXenes have also been studied as an emerging material for application in water purification, i.e., capacitive deionization, membranes, and adsorption [36–45].

This review is focused on the evaluation of MXenes and MXene-based composites for applications in desalination. The desalination potential of MXenes is portrayed in detail with an emphasis on ion-sieving membranes, capacitive deionization, and solar desalination. The key challenges and issues allied with the synthesis and applications of MXenes and MXene-based composites in desalination are highlighted. Future research directions are provided to guarantee the efficient synthesis and applications of MXenes in desalination.

## Desalination Potential of MXenes

MXenes and their composites have demonstrated a substantial potential in numerous environmental applications such as adsorption, membrane separations, and capacitive deionization. MXenes are promising aspirants for water purification membranes owing to their hydrophilicity, high surface area, and excellent mechanical and electrical properties [46–48]. MXenes and MXene-based composites are favorable candidates in desalination applications. MXene (Ti_3_C_2_T_*x*_) has a low contact angle of 21.5°, and it exhibited excellent stability in water even after vigorous shaking that makes them an ideal candidate for desalination applications [49].

### Ion-Sieving Membranes

MXene nanosheets can be employed for rejection of size- and charge-selective rejection of molecules and ions either in freestanding or supported membranes. Performance evaluation of different MXene membranes for potential applications in desalination is presented in Table 1. Molecules can be separated via size exclusion through the stacked layers and by interaction with the charged MXene layers. Smaller ions from water can be eliminated due to constrained interlayer spacing of MXenes. The water flux through MXene (Ti_3_C_2_T_*x*_)-based membranes was high, and the membranes exhibited excellent separation potential for various ions based on charge and hydration radius of the ion [36, 50].

**Table 1:** Ion sieving performance evaluation of different MXene membranes

Membrane	Rejection (%)	Permeability (L/m^2^ h bar)	Remarks	References
MXene-derived membranes supported on α-Al_2_O_3_	Na_2_SO_4_: 75.9 MgSO_4_: 67.3 NaCl: 55.3 MgCl_2_: 46.1	22.4 L	The interlayer spacing between nanosheets of MXene-derived membranes can be adjusted by regulating the sintering temperature The interlayer spacing decreased with an increase in temperature At temperature > 400 °C, the oxidation of Ti_3_C_2_T_*x*_ nanosheets into TiO_2_ NPs occurs and the ion retention reduced	[54]
Ti_3_C_2_T_*x*_ membrane	–	38	The flux through MXene membrane declined with increasing thickness until reaching a steady-state value at higher thickness The permeation was found dependent on both the cations’ size and charge Better selectivity is shown toward charged metal cations and dye cations of different sizes than GO	[36]
Ag@MXene composite membrane	MG: 92.32 RhB: 79.93 BSA: 100 NaCl: 25.8 MgCl_2_: 41.3 AlCl_3_: 49.5	420	Ag@MXene membrane demonstrated higher water flux than pristine MXene membranes Ag@MXene composite membrane also revealed enhanced bactericidal characteristics and moderate salt rejection	[51]
MXene membrane supported on anodic aluminum oxide (AAO) substrate	BSA: 100 RB, Rhodamine B: 85 EB, Evans Blue: 90 Cytochrome C: 97	1084	Membrane exhibited high water flux (> 1000 L/m^2^ h bar) and promising rejection rate (> 90 %) for molecules with sizes larger than 2.5 nm The improved water permeation can be attributed to the additional nanochannels formed in the MXene membrane after deposition on AAO support	[53]
Self-cross-linked MXene (Ti_3_C_2_T_*x*_) membranes	NaCl: 98.6	0.0515	Self-cross-linked MXene membrane exhibited excellent stability and anti-swelling property Membrane demonstrated improved performance of the ion exclusion compared to pristine MXene	[57]
Ti_3_C_2_T_*x*_	MB: 98.0 ± 0.9	10.6	Positive and negative voltages decreased and increased rejection through MXene membranes, respectively Exclusion of ions from the Ti_3_C_2_T_*x*_ membranes by the application of electrical voltage provides a facile method to regenerate the MXene membranes	[59]
Ti_3_C_2_T_*x*_/poly(vinyl alcohol (PVA)	MB: 97.2 ± 0.8	7.4		
MXene slit membranes (MD simulation)	Monovalent salt ions: 100	400	The material’s inherent interaction parameters, hydrophobicity, and shape of the slits were found to affect the desalination performance	[55]

**Fig. 2:**
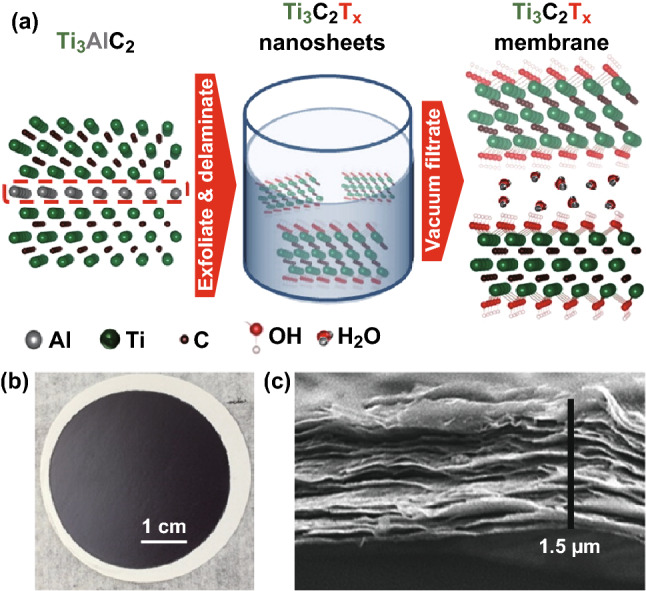
**a** A schematic of the synthesis of a Ti_3_C_2_T_*x*_ membrane, **b** photograph, and **c** SEM image of cross-sectional of the Ti_3_C_2_T_*x*_ membrane. Reprinted with the permission from Ref. [36]. Copyright © (2015) American Chemical Society

Ren et al. [36] fabricated the Ti_3_C_2_T_*x*_ membrane by a vacuum-assisted filtration (VAF) method (Fig. 2) and reported the selective sieving of various cations through the membrane. The thickness of the membrane played a crucial role in water permeation through the membrane. The flux through the MXene membrane declined with increasing thickness until reaching a steady-state value at higher thickness. MXene membrane exhibited an ultrafast water flux ~ 38 L (m^2^ h bar)^−1^ at the membrane thickness of 1500 nm.

**Fig. 3:**
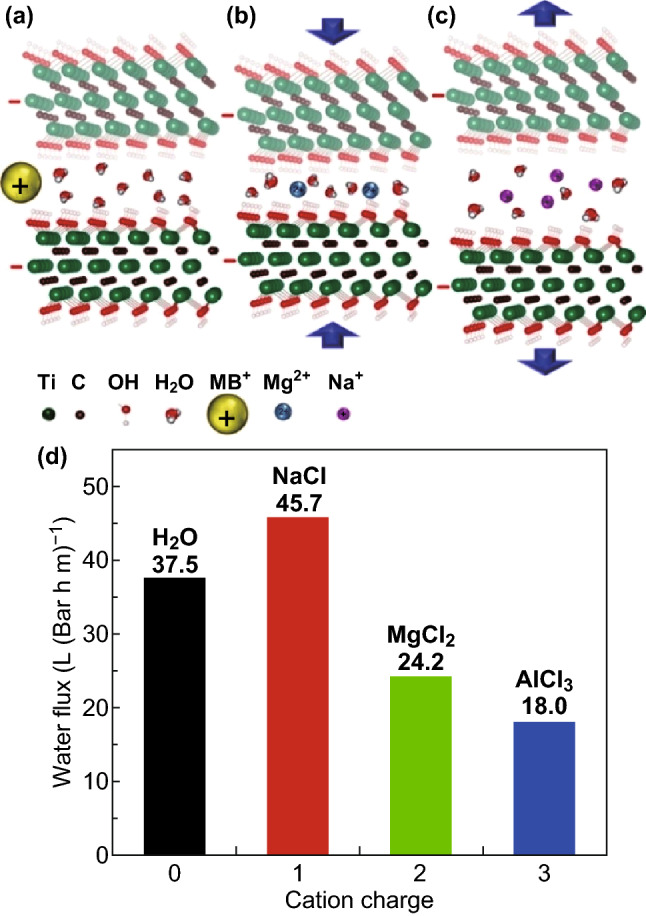
Schematics of the permeation of cations through a Ti_3_C_2_T_*x*_ membrane and total rejection of large MB^+^. **a** Cations of radii > 6.4 Å cannot penetrate into interlayer spacing. **b** Shrinkage of spacing between Ti_3_C_2_T_*x*_ layers due to the intercalation of smaller multiple-charged cations that result in slower permeation. **c** Expansion of Ti_3_C_2_T_*x*_ interlayer spacing due to the intercalation of small single-charged cations that result in faster flow. **d** The flux of salt solutions and water through the Ti_3_C_2_T_*x*_ membranes versus cation’s charge. Reprinted with permission from Ref. [36]. Copyright © (2015) American Chemical Society

As depicted in Fig. 3, the interlayer distances of the Ti_3_C_2_T_*x*_ membranes expedite the fast flow of water. The permeation rates of alkali, alkaline earth (Li^+^, Na^+^, K^+^, Mg^2+^, and Ca^2+^), transition and other metal (Ni^2+^ and Al^3+^), and methylthioninium^+^ (MB^+^) dye cations followed the order: Na^+^ > Li^+^ > K^+^ > Ca^2+^ > Ni^2+^ > Mg^2+^ > Al^3+^ ≫ MB^+^. The permeation was found dependent on both the cations’ size and charge. Single-charged Na^+^ or K^+^ ions were found to intercalate readily into the MXene membrane compared to the double-charged Mg^2+^ ions and bulky ions such as Al^3 +^. MXene membranes show better selectivity toward charged metal cations and dye cations of different sizes than graphene oxide [36].

Cations with larger radii than the interlayer distance (~ 6.4 Å) are debarred based on size. Cations with radii smaller than the interlayer distance can penetrate into the interlayer capillary pathways and increase the interlayer spacing. However, when abundant ions are intercalated, the electrostatic attraction between the charged ions and negatively charged MXene surface results in shrinkage of the layers. Finally, single-charged ions (Na^+^) can pass through the membrane and are attracted to each side of MXene layers, forming an electric double layer on the surface and causing expansion of Ti_3_C_2_T_*x*_ interlayer spacing. The actuation mechanism explains the cations permeation and high flux of Na^+^ due to increasing the interlayer spacing. On the other hand, the low flux of Mg^2+^ and Al^3+^ is attributed to the shrinkage interlayer spacing.

**Fig. 4:**
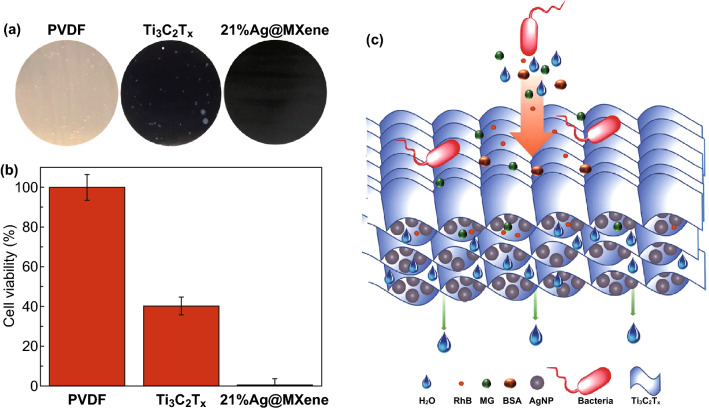
Antibacterial activity of PVDF (control), MXene (Ti_3_C_2_T_*x*_), and 21% Ag@MXene membranes: **a** photographs of *E. coli* cell growth incubated at 35 °C for 24 h. **b** Cell viability measurements of *E. coli*. **c** Schematic structure and mechanism of rejection of the 21% Ag@MXene composite membrane. Reprinted with permission from Ref. [51]. Copyright © (2019) Royal Society of Chemistry

The water flux through the pristine MXene (Ti_3_C_2_T_*x*_) was significantly enhanced from ~ 118 to ~ 420 L (m^2^ h bar)^−1^ by modifying MXene with Ag nanoparticles (Ag@MXene) [51]. The enhanced water flux after modification is due to a decrease in contact angle and creation of a slit interspacing of 1–4 nm between the MXene nanosheets providing excess nanopores in the Ag@MXene membranes. The Ag@MXene composite membrane also revealed enhanced bactericidal characteristics compared to pristine MXene and polyvinylidene difluoride (PVDF)-based membranes, by exhibiting 99% *E. coli* growth inhibition as shown in Fig. 4. The Ag@MXene composite membrane showed moderate rejection of salts that might be due to their open structure after modification.

**Fig. 5:**
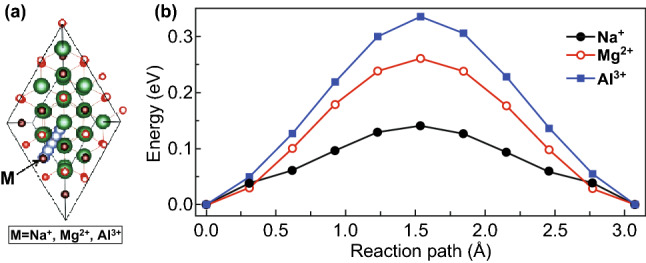
**a** Considered ion migration pathway and **b** corresponding energy profiles of Na^+^‏, Mg^2+^‏, and Al^3+^. Reprinted with permission from Ref. [52]. Copyright © (2019) AIP Publishing LLC

The surface charges of the membrane also affect the ion sieving performance, as confirmed by density functional theory calculations [52]. Ions of different charge states behave differently due to the difference in energy barriers for the intercalation, and spacing between the MXene can be expanded or contracted that affects the ion sieving rate. The energy barrier width increased, while the interlayer space decreased, with increasing the charge of the cations, as shown in Fig. 5 [52]. These findings suggest that ion sieving performance of MXene membranes can be enhanced by modifying the surface charges.

**Fig. 6:**
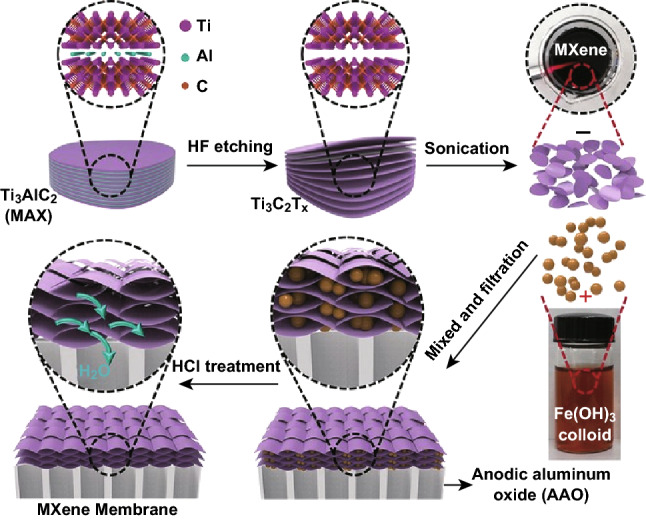
Schematic of MXene membrane preparation via HF etching and intercalation by using Fe(OH)_3_ colloidal solution. Reprinted with permission from Ref. [53]. Copyright © 2017 John Wiley & Sons, Inc

Ding et al. [53] prepared MXene nanosheets by etching the Ti_3_AlC_2_ particles by HF solution to generate Ti_3_C_2_T_*x*_ powder followed by sonication‐assisted exfoliation, as shown in Fig. 6. The MXene nanosheets were intercalated by using Fe(OH)_3_ colloidal solution that created the expanded nanochannels. The MXene membrane was obtained by a vacuum filtration process followed by a hydrochloric acid solution (HCl) treatment to eradicate the Fe(OH)_3_ nanoparticles.

The highest water flux (> 1000 L (m^2^ h bar)^−1^ was reported by Ding et al. for the MXene membrane supported on anodic aluminum oxide (AAO) substrate [53]. The membrane also demonstrated a promising rejection rate (> 90%) for molecules with sizes larger than 2.5 nm. The enhanced water flux can be ascribed to the surplus nanochannels formed in the MXene membrane after deposition on AAO support.

**Fig. 7:**
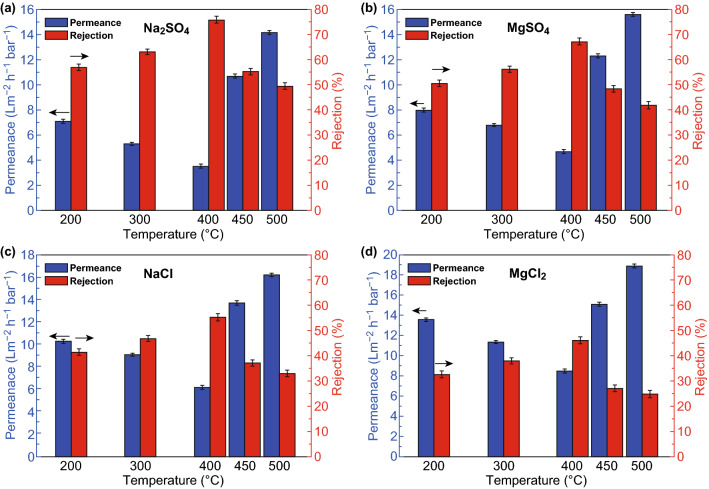
**a**–**d** Rejections and salt permeances of the MXene membranes. Reprinted with permission from Ref. [54]. Copyright © (2019) Elsevier B.V

Membrane permeation performance can be enhanced further by tuning of membrane properties. The interlayer spacing between stacked neighboring nanosheets of ultrathin MXene-derived membranes supported on α-Al_2_O_3_ was adjusted by regulating the sintering temperature [54]. MXene membranes were synthesized by depositing the MXene dispersion on the inner surface of the porous ceramic substrate. The prepared membranes were dried and calcinated at different temperatures ranging from 200 to 500 °C in air. The interlayer spacing decreased with an increase in temperature probably due to de-functionalization (–OH) occurred within the MXene film at high temperature and moisture loss. At temperature > 400 °C, the oxidation of Ti_3_C_2_T_*x*_ nanosheets into TiO_2_ NPs occurs and the ion retention reduced due to change in filtration mode from interlayer transport pathways to longitudinal nanochannels. The higher retention was obtained for membrane sintered at 400 °C, while the salt rejection of various ions followed the order: Na_2_SO_4_ > MgSO_4_ > NaCl > MgCl_2_, whereas the water and salt permeation were greater for membrane calcinated at 200 °C, as depicted in Fig. 7.

The rejection and permeation performance of MXene slit membranes were predicted by molecular dynamics (MD) simulation [55]. The material’s inherent interaction parameters, hydrophobicity, and shape of the slits were found to affect the desalination performance. Mixed matrix membrane comprising MXene and P84 copolyimide demonstrated high flux (268 L (m^2^ h)^−1^) at 0.1 MPa and excellent solvent resistance that suggests their potential applications as nanofiltration membranes [56]. The incorporation of the inorganic additive has a positive impact on water permeability and solvent resistance.

**Fig. 8:**
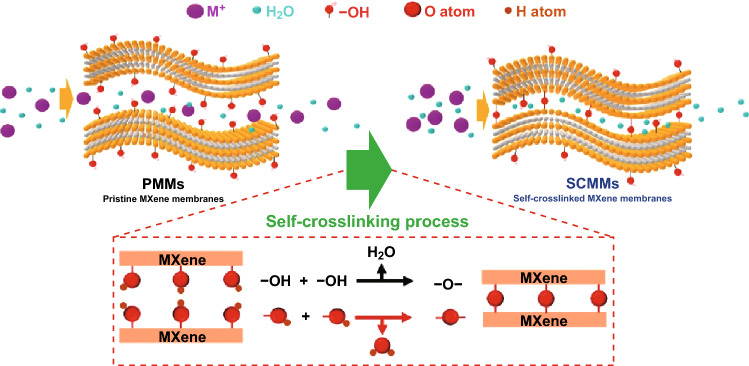
Self-cross-linking process of the MXene membranes. Reprinted with permission from Ref. [57]. Copyright © (2019) Royal Society of Chemistry

Lu et al. [57] synthesized the self-cross-linked MXene membrane through a facile thermal treatment. Self-cross-linking reaction (−OH^+^ −OH = − O^−^ + H_2_O) resulted in the formation of Ti–O–Ti bonds between the MXene nanosheets, as depicted in Fig. 8. The resulting membrane exhibited excellent stability and anti-swelling property in addition to the improved performance of the ion exclusion (98.6% of NaCl) compared to pristine MXene. The stability of the self-cross-linked MXene membrane compared to polymeric membranes, under acid/base conditions, advocates their promising potential in desalination.

Solvent-resistant polymer–Ti_3_C_2_T_*x*_ nanofiltration membranes exhibited excellent permeation performance for alcohol molecules by providing additional pathways along nanosheet surface using –OH as an adsorption site [58]. The membrane also demonstrated good solvent resistance and rejection abilities, in addition to improved mechanical and thermal stability. This endorses the probable applications of MXene and polymer composite membranes in desalination.

**Fig. 9:**
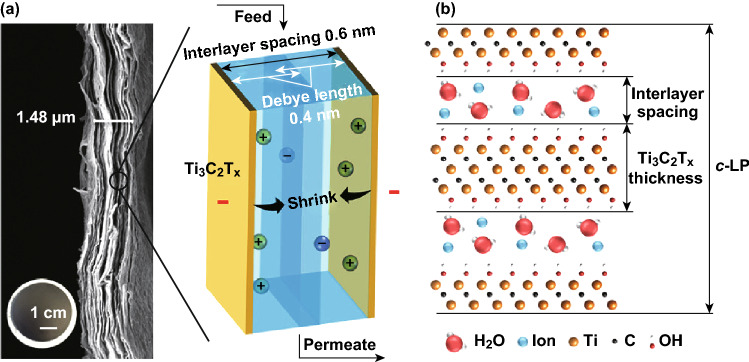
**a** Cross-sectional SEM image of a Ti_3_C_2_T_*x*_ membrane with a schematic showing the interaction between Ti_3_C_2_T_*x*_ layers and ions within their EDLs when the Ti_3_C_2_T_*x*_ membrane is connected to a negative voltage. **b** Molecular model of Ti_3_C_2_T_*x*_ showing two layers of water with ions between the layers. The *c* lattice parameter (*c*-LP) calculated from XRD is two Ti_3_C_2_T_*x*_ flake thicknesses and two interlayer spacings. Reprinted with permission from Ref. [59]. Copyright © (2018) American Chemical Society

Ren et al. [59] reported the voltage-gated ions sieving through the nanochanneled Ti_3_C_2_T_*x*_ membrane. The rejection of MgSO_4_ and NaCl enhanced by applying a negative electrical potential (−0.6 V) to the Ti_3_C_2_T_*x*_ membrane and inhibited after applying a positive potential due to electrostatic repulsion between the cations and charged MXene layers. The voltage-gated rejection through electronically conductive membranes represents a promising breakthrough approach to improve the elimination of various ions. Figure 9 illustrates orderly stacked 2D nanochannels across the structure of a Ti_3_C_2_T_*x*_ membrane and channel (interlayer spacing) between two MXene sheets.

### Capacitive Deionization (CDI)

CDI is an energy-efficient electrochemical process in which anions and cations are separated based on their charge and deposited on positively charged anode and negatively charged cathode, respectively. The selection of electrode materials is critical in the CDI process, and an ideal electrode must have a high surface area, good electrical conductivity, and high stability. Owing to their superior pseudocapacitive properties, MXenes have emerged as novel electrode materials for CDI applications [60–67]. The MXene-based CDI electrode proved a marvelous adsorption capacity for both cations and anions simultaneously due to their high electrical conductivity, hydrophilicity, and tunable surface [39, 62, 68]. Table 2 summarizes the desalination performance of MXene electrodes in CDI.

**Table 2:** Desalination performance of MXene electrodes in CDI

Electrode material	Surface area (m^2^ g^−1^)	NaCl concentration (mM)	Cell voltage (V)	Salt removal capacity (mg g^−1^)	Remarks	References
Ti_3_C_2_ MXene	6	5	1.2	13 ± 2	MXene CDI electrodes demonstrated excellent performance in 30 cycles The adsorption of ions onto the electrode occurs via ion intercalation instead of double-layer formation	[39]
Porous Ti_3_C_2_T_*x*_ MXene	293	10,000 mg L^−1^	1.2	45	MXene electrode demonstrated 12 times higher ion adsorption capacity than other carbon-based electrodes and excellent cycling stability (up to 60 cycles)	[62]
Mo_1.33_C-MXene	1	5/50/600	0.8	5/9/15	Incorporation of carbon nanotubes enhanced the desalination performance of MXene electrode Low energy requirement compared to traditional carbon electrode	[68]
Ar plasma-modified Ti_3_C_2_T_*x*_	–	500 mg L^−1^	1.4	26.8	Ar plasma modification of MXene nanosheets resulted in the increased interlayer distance between the sheets Electrode showed good regeneration ability and reproducible results	[60]
Porous nitrogen-doped MXene sheets (N–Ti_3_C_2_T_*x*_)	368.8	5000 mg L^−1^	1.2	43.5 ± 1.7	Nitrogen doping significantly enhances the surface area and desalination performance of MXene	[64]
LiF/HCL-etched Ti_3_C_2_T_*x*_ MXene	2.1	585	1.2	67.7	The LiF/HCl etching resulted in the increased interlayer spacing of Ti_3_C_2_T_*x*_ and enhanced desalination capacity	[65]
Preconditioned Ti_3_C_2_T_*x*_ MXene	–	10	− 1.2 (discharge potential (V)	9.19	Operating conditions such as flow rate, half-cycle length (HCL), and discharge potential affect the desalination performance of electrode	[67]

**Fig. 10:**
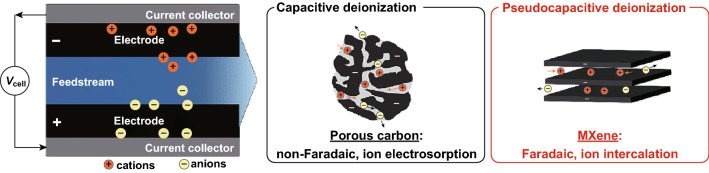
Concept of electrochemical water desalination by non-faradaic (electrostatic) ion electrosorption at the surface of electrode materials. Reprinted with permission from Ref. [39]. Copyright © (2019) Royal Society of Chemistry

Srimuk et al. [39] introduced the MXene-based CDI electrode for the first time by casting Ti_3_C_2_ onto the porous separator of the CDI cell. MXene CDI electrodes demonstrated excellent performance in 30 cycles with a salt adsorption capacity of 13 ± 2 mg g^−1^ (1.2 V cell voltage in 5 mM NaCl saline solution). The adsorption of ions onto the electrode occurs via ion intercalation instead of double-layer formation, as schematically depicted in Fig. 10.

Desalination performance of the MXene electrode was further enhanced by entangling MXene with carbon nanotubes to form a binder-free CDI electrode [68]. The resultant Mo_1.33_C-MXene electrode presented a charge efficiency of ~ 97% and desalination capacity of 15 mg g^−1^ in 600 mM NaCl. The prepared electrode has the advantage of low energy requirement compared to traditional carbon electrodes. The excellent desalination performance of the electrode in the absence of ion-exchange membranes provides a strong base for its applications in future desalination technology. Incorporation of polymer binders and carbon nanotubes into MXenes results in composite with enhanced electrical conductivity, mechanical strength, and tremendous flexibility that can be exploited for desalination applications [69, 70]. The addition of polymer or nanomaterials also influences the interlayer spacing [70].

**Fig. 11:**
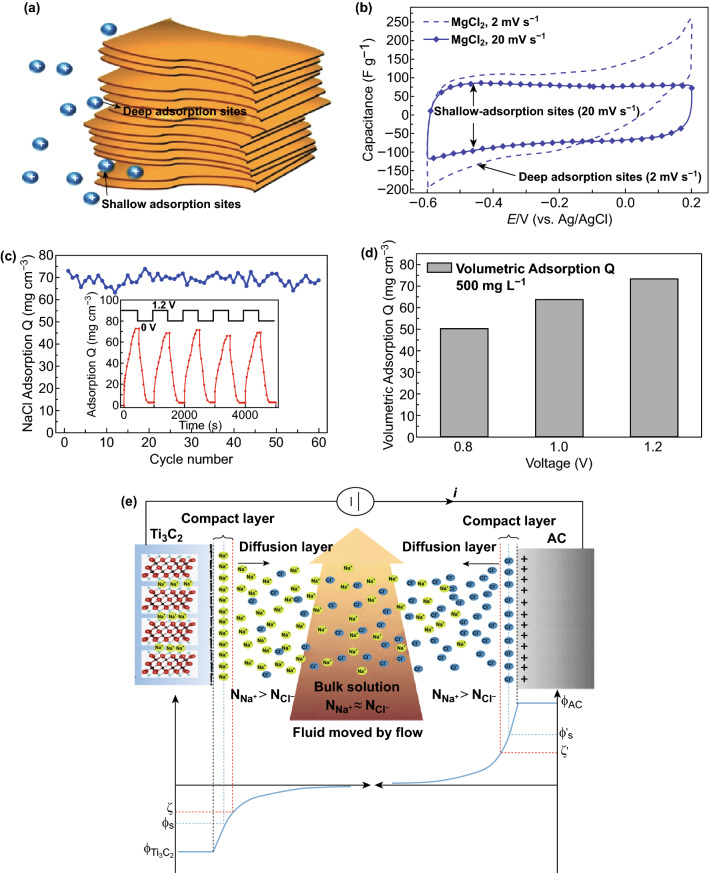
**a** Schematic illustration of multilayered Ti_3_C_2_T_*x*_ particle containing shallow and deep adsorption sites near the gap opening and in its interior, respectively, **b** Capacitance of Ti_3_C_2_T_*x*_/carbon black–PTFE laminate in a Swagelok cell measured at scan rates of 2 and 20 mV s^−1^ (dash line and symbols, respectively) in 1 M MgCl_2_ related to deep and shallow adsorption sites, respectively. Reprinted with permission from Ref. [66]. Copyright © (2015) John Wiley & Sons, Inc. **c** Electroadsorption and regeneration cycles of porous Ti_3_C_2_T_*x*_ electrode in 500 mg L^−1^ NaCl solution. **d** Electroadsorption capacitance of porous Ti_3_C_2_T_*x*_ at varied voltage in 500 mg L^−1^ NaCl solution. Reprinted with permission from Ref. [62]. Copyright © (2018) Elsevier Inc. **e** Schematics of the CDI process. Reprinted with permission from Ref. [60]. Copyright © (2018) Elsevier B.V

Levi et al. [66] demonstrated that two types of cationic adsorption sites (shallow and deep) are present in the interlayer gaps of the Ti_3_C_2_T_*x*_ layers. The swelling of the multilayered Ti_3_C_2_T_*x*_ occurs spontaneously when in contact with the solution at open-circuit potential, and adsorbed cations are electrochemically inserted between partially swollen Ti_3_C_2_T_*x*_ layers. The deep adsorption exists in the particle’s interior, while the shallow adsorption sites are present near the edges of the multilayer particles that are water-rich, as depicted in Fig. 11a. Ion accumulation on the deep adsorption is possible by increasing the cathodic current to − 0.6 V at slow scan rates after a large number of cycles, and/or by slowly charging and discharging (Fig. 11b). Electroadsorption performance of porous Ti_3_C_2_T_*x*_ electrode in NaCl solution is presented in Fig. 11c, d, while Fig. 11e shows schematics of the CDI process.

**Fig. 12:**
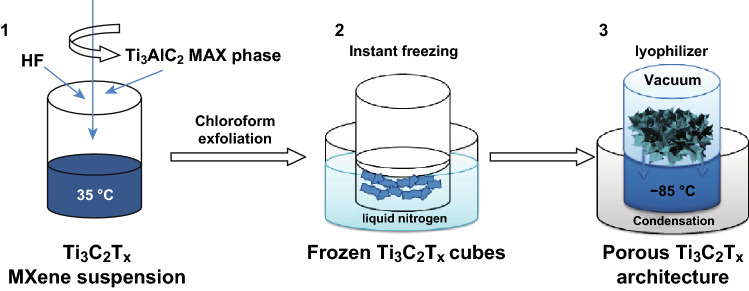
Synthetic procedure for the porous Ti_3_C_2_T_*x*_ architecture. Reprinted with permission from Ref. [62] Copyright © (2018) Elsevier Inc

Porous Ti_3_C_2_T_*x*_ architectures for CDI applications are prepared by chloroform exfoliation of Ti_3_C_2_T_*x*_ followed by freezing the resultant suspension in liquid nitrogen and vacuum-drying the frozen Ti_3_C_2_T_*x*_ cubes [62]. The process is illustrated schematically in Fig. 12.

Aerogel-like porous MXene electrode material unveiled excellent capacitive deionization performance with 12 times higher ion adsorption capacity than other carbon-based electrodes [62].

The porous MXene electrode exhibited excellent cycling stability (up to 60 cycles) with the reported ion adsorption capacity of 45 mg g^−1^ in a 10,000 mg L^−1^ salt solution. The ion intercalation into the interlayer space of the MXene also contributes to salt adsorption in addition to capacitive storage. The superior performance of the electrode was attributed to the high electrical conductivity, high surface area, well-defined porous structure, and hydrophilic nature of the Ti_3_C_2_T_*x*_.

The porous Ti_3_C_2_T_*x*_ CDI electrode showed excellent cyclic stability up to 60 cycles (Fig. 11c), and the highest electroadsorption capacitance reaches within ~ 450 s. The electrode demonstrated good reversibility and was easily regenerated when the applied voltage dropped back to 0 V. The electroadsorption capacities increased with an increase in voltage from 0.8 to 1.2 V, as shown in Fig. 11d.

Doping of MXene sheets with nitrogen yields a porous electrode (N–Ti_3_C_2_T_*x*_) with high surface and promising desalination performance [64]. Nitrogen doping significantly enhances the surface area of MXene to 368.8 m^2^ g^−1^ that is the highest value reported in the literature for any MXene-based electrode. N–Ti_3_C_2_T_*x*_ demonstrated an average salt adsorption capacity of 43.5  ±  1.7 mg g^−1^ under 1.2 V in 5000 mg L^−1^ NaCl solution. The electrode shows good stability over 24 CDI cycles. The etching process also influences the desalination characteristics of MXene electrode. Ma et al. [65] employed the LiF/HCL etching method to prepare a freestanding Ti_3_C_2_T_*x*_ MXene electrode without any binder and evaluated its desalination performance. The LiF/HCl etching resulted in the increased interlayer spacing of Ti_3_C_2_T_*x*_ and enhanced desalination capacity. The electrode exhibited a desalination capacity of 68 mg g^−1^ at 1.2 V for NaCl concentration of 585 mg L^−1^.

Ar plasma modification of MXene nanosheets resulted in the increased interlayer distance between the sheets and hence improved desalination performance [60]. The surface of Ti_3_C_2_T_*x*_ was modified to introduce amorphous carbon and anatase TiO_2_ layer using Ar plasma treatment. The desalination performance of the electrode was evaluated using 500 mg L^−1^ NaCl solution in the voltage range of 0.8–1.6 V, as shown in Fig. 11e. The maximum removal capacity of 26.8 mg g^−1^ was obtained at 1.2 V. The Ti_3_C_2_-based electrode showed good regeneration ability and reproducible results for several cycles of electrosorption and desorption.

Desalination performance of MXene electrode is influenced by the operating conditions such as flow rate, half-cycle length (HCL), and discharge potential [67]. Agartan et al. [67] reported that salt adsorption rate and capacity increased by 152% at lower discharge potentials and decreased at faster flow rates. Likewise, half-cycle length decreased salt adsorption rate by 54% and capacity by 32%. Preconditioned MXene electrodes exhibited better volumetric performance than activated carbon cloth electrodes owing to their hydrophilicity and high electrochemical activity.

### Solar Desalination

MXenes have superb light-to-heat conversion efficiency that makes them an ideal applicant for application in solar-based desalination [71]. The photothermal water evaporation capability of MXenes is yet another energy-efficient characteristic of these fascinating 2D materials. Table 3 enlists the solar evaporation performance of MXene membranes.

**Table 3:** Solar evaporation performance of MXene membranes

Material	Efficiency (%)	Evaporation rate [kg (m^2^ h)^−1^]	Remarks	References
Ti_3_C_2_ MXene	84	1.373	The MXene, Ti_3_C_2_ demonstrated 100% light-to-heat conversion efficiency	[71]
Ti_3_C_2_ MXene	71	1.31	The hydrophobic MXene membrane exhibited excellent solar evaporation potential Not suitable for long-term solar desalination due to poor salt-blocking after an elongated period Extraordinary stability (> 200 h) under actual seawater conditions	[72]
VA-MXA	87	1.46	The hydrophobic upper layer absorbed light and the hydrophilic bottom layer pumped water The high water evaporation rate is attributed to the strong capillary pumping and fast water diffusion through the vertically aligned channels of the VA-MXA	[74]

**Fig. 13:**
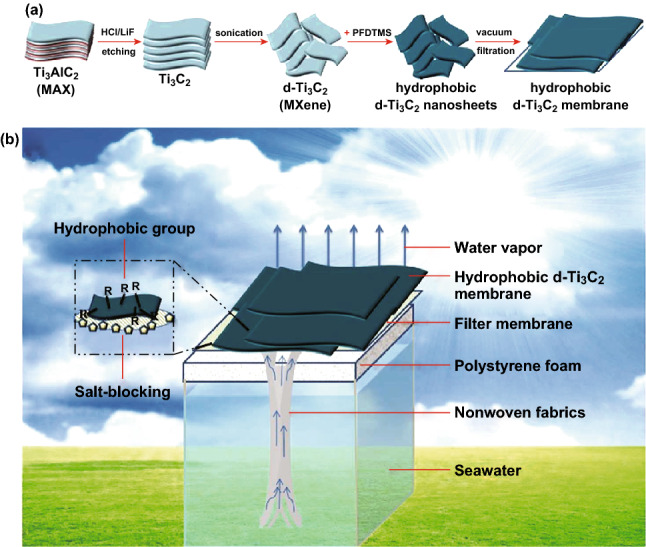
Schematic illustration of **a** fabrication process of the hydrophobic d-Ti_3_C_2_ membrane, and **b** the hydrophobic d-Ti_3_C_2_ membrane-based solar desalination device. Reprinted with permission from Ref. [72]. Copyright © (2019) Royal Society of Chemistry

Zhao et al. [72] reported the synthesis and solar desalination potential of the hydrophobic MXene membrane. The delaminated Ti_3_C_2_ (d-Ti_3_C_2_) was obtained by HCl/LiF etching from the MAX phase, followed by vacuum deoxidation and ultrasonication, as shown in Fig. 13. The hydrophobic membranes were obtained by surface modification of the d-Ti_3_C_2_ with trimethoxy(1H,1H,2H,2H-perfluorodecyl)silane (PFDTMS) [72].

**Fig. 14:**
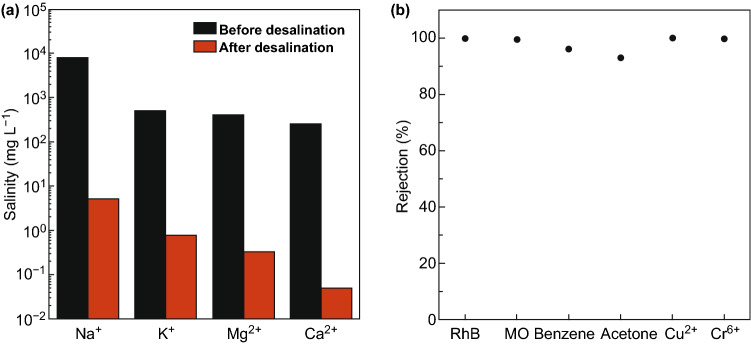
**a** Measured salinity of four primary ions before and after solar desalination. **b** Organic and heavy metal ion rejection performance. Reprinted with permission from Ref. [72]. Copyright © (2019) Royal Society of Chemistry

The hydrophobic MXene membrane obtained after PFDTMS modification was employed in a solar evaporation device that was self-floated on the seawater. The membrane achieved a solar steam conversion efficiency of 71%, the solar evaporation rate of 1.31 kg m^2^ h^−1^, and stability under high salinity conditions over 200 h under one sun. The rejection rate for the four primary ions (Ca^2+^, Mg^2+^, Mg^2+^, and Na^+^) was over 99.5%, while for organic dyes and heavy metals, nearly 100% rejection rate was attained, as shown in Fig. 14. These membranes are not appropriate for long-term solar desalination applications due to poor salt-blocking after an elongated period.

MXene coating improved the antifouling and photothermal characteristics of the PVDF (polyvinylidene difluoride) in a solar-assisted direct contact membrane distillation system [73]. MXene-coated membrane demonstrated around 56% reduction in flux decline and a 12% drop in heater energy input per unit volume of distillate. However, MXene-coated membranes exhibited lesser fluxes due to the presence of an additional coating layer.

**Fig. 15:**
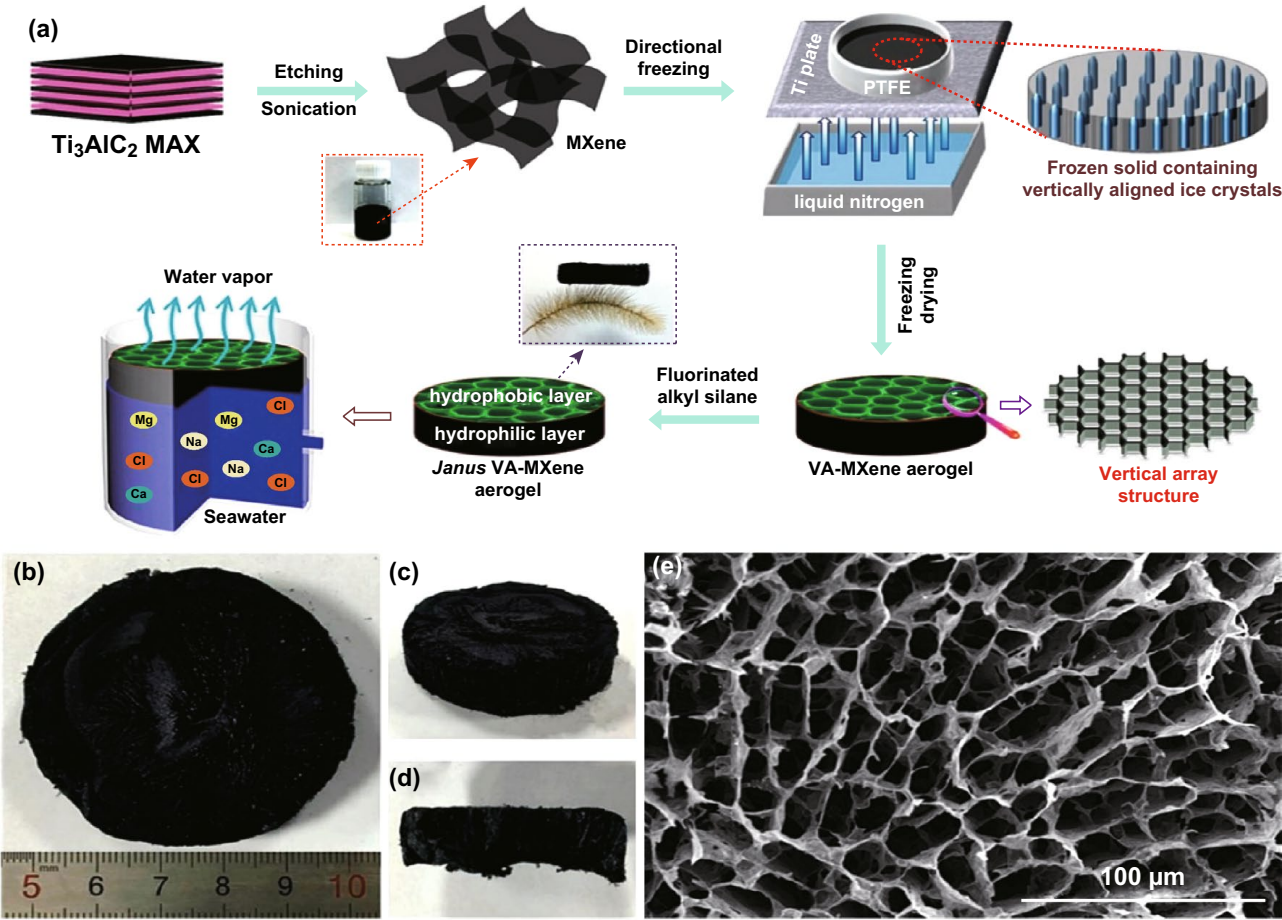
**a** Fabrication process of a Janus VA-MXA with vertically aligned channels. **b** Digital photograph of the top view of the as-prepared Janus VA-MXA. **c** Photograph of the side view. **d** Photograph of the fracture face. **e** SEM images of the upper layer of prepared Janus VA-MXA. Reprinted with permission from Ref. [74]. Copyright © (2019) American Chemical Society

Zhang et al. [74] reported the desalination performance of vertically aligned Janus MXene aerogel (VA-MXA) with two layers, i.e., hydrophilic (at the bottom) and hydrophobic (at the top). The process of VA-MXA synthesis is presented in Fig. 15. MXene obtained from the Ti_3_AlC_2_ phase is frozen by liquid nitrogen under Ar protection in a polytetrafluoroethylene (PTFE) mold with a Ti plate. The freezing process yields a black frozen material consisting of ice crystals surrounded by Ti_3_C_2_ nanosheets. A vertically aligned framework of the Ti_3_C_2_ nanosheets is obtained by removing ice crystals via vacuum freeze-drying. The freestanding VA-MXA was placed in a ring-shaped sponge mold, and the hydrophobic layer was formed by floating the sponge on fluorinated alkyl silane under vacuum conditions, followed by drying under Ar environment.

The hydrophilic bottom layer of VA-MXA pumped water and the hydrophobic upper layer absorbed light. The salts crystallized on the hydrophilic bottom layer are quickly dissolved due to continuous pumping of water. The VA-MXA demonstrated an excellent water evaporation rate of 1.46 kg (m^2^ h) ^−1^ and conversion efficiency of ~87%. The high water evaporation rate is attributed to the strong capillary pumping and fast water diffusion through the vertically aligned channels of the VA-MXA.

### Pervaporation Desalination

**Fig. 16:**
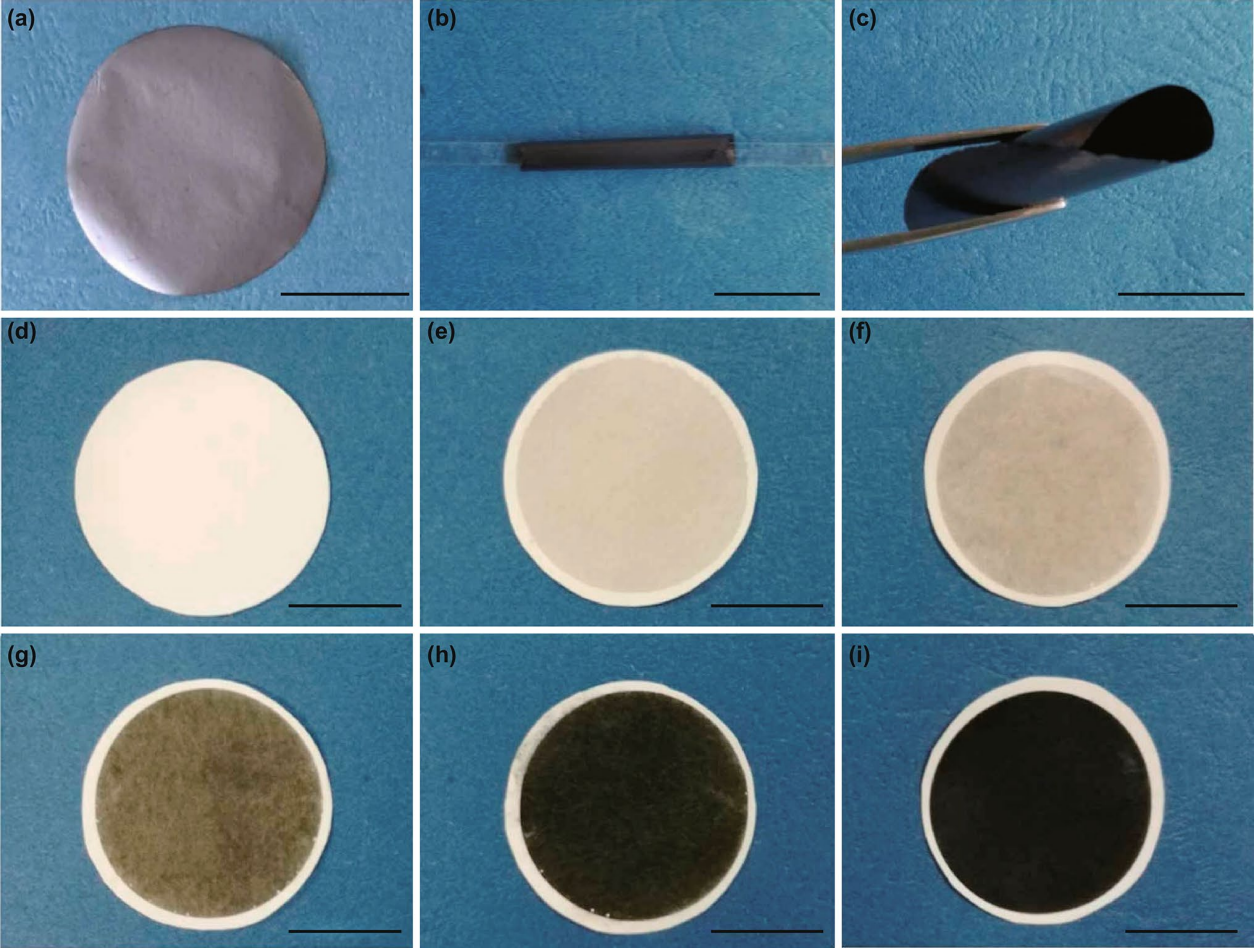
Digital images of membranes **a** freestanding MXene membrane, **b, c** folded MXene membrane, **d–i** MXene membrane with different deposition amount, i.e., (0, 3.662, 7.325, 18.31, 36.62, 73.25) × 10^−6^ g cm^−2^. Reprinted with the permission from Ref. [75]. Copyright © (2017) Elsevier B.V

Pervaporation desalination is a combination of water diffusion through a membrane followed by its evaporation into the vapor phase on the other side of the membrane. MXene/PAN composite and freestanding MXene membranes were prepared by vacuum filtration of the MXene suspension through the polymeric substrate, as shown in Fig. 16 [75]. A specific amount of MXene nanosheets were deposited on PAN substrate to prepare MXene/PAN composite (Fig. 16d–i), while freestanding MXene membranes were prepared via similar approach by using polycarbonate (PC), followed by exfoliation of MXene from substrates after drying for 24 h (Fig. 16a–c). As the amount of MXene nanosheets increases, the thickness of the MXene layer increases and its color varies from light green to dark green.

A home-made apparatus was employed to measure the pervaporation desalination performance of the MXene membranes. The membranes consist of stacked atomic-thin MXene nanosheets (~ 60 nm) supported on commercial PAN ultrafiltration membranes exhibiting tremendous potential for pervaporation desalination [75]. The membranes rejected 99.5% salt and exhibited a water flux of 85.4 L (m^2^ h) ^−1^ at 65 °C. The MXene membrane also demonstrated great desalination performance for synthetic seawater compared to the other membranes reported in the literature. One hurdle associated with pervaporation desalination is the intensified use of energy. However, with the utilization of renewable energy or energy from waste sources, a significant improvement in energy efficiency is expected.

## Challenges and Future Prospects

Although MXenes have emerged as fabulous materials for potential applications in desalination, there are still plentiful challenges and baffling disputes that need to be addressed to fully exploit their remarkable properties [3, 9, 13, 17, 18, 76]. MXenes are mainly synthesized through a top-down route, and very limited literature is available on the synthesis of MXenes using a bottom-up approach [13, 77]. A significant consideration is desired in this research drift to explore the novel bottom-up methods of MXene synthesis with more control of product characteristics.

The MXenes family can be further extended by exploring the synthesis of MXenes from other potential MAX phases [78]. Furthermore, the same technique for MXene synthesis may be applied to produce more 2D materials from MAX-like phases such as MAB. Another hurdle for researchers to be overcome is the requirement of sub-zero temperatures for the storage of MXenes [79]. It is essential to develop an effective technique for storing MXene solution for a long time without oxidizing.

The traditional method for the synthesis of MXenes using hazardous HF is associated with serious health and environmental concerns. The replacement of HF with green or less toxic chemicals can assure an environment-friendly technique for the synthesis of MXenes. There are some attempts in recent times to substitute HF with less toxic chemicals [8, 80, 81]. However, more attention is required to advance research in this direction. Furthermore, the difficulty in the synthesis of MXenes with uniform and pure surface termination is another obstacle in their practical applications [82].

Another major challenge is the high cost and low yield of MXenes production [17, 18]. Currently, MXenes are mainly produced at the laboratory scale with a small yield. The design of a cost-effective, efficient, and environment-friendly system for the large-scale production of MXenes will be helpful to further advance research in this field and will open a new door of possible applications of MXenes on a commercial scale. It is expected that the cost will be comparatively low for large-scale production.

One more crucial challenge is the need for life cycle analysis and assessment of potential toxic effects of MXenes and MXene-based nanomaterials [18, 83, 84]. Still, studies on the potentially toxic effects of MXenes are limited. With the rapid deployment of MXenes in various applications, it is necessary to fully investigate its lethal effects on the environment, human health, and other organisms. Surface modification of MXenes could be effective in improving its stability, biocompatibility, and recyclability and reducing cytotoxicity. The aggregation of MXenes is also an issue that reduces the adsorption capability and surface area of these 2D materials. The surface chemistry of MXenes and their influence on the removal of pollutants must be further explored to fully understand the removal mechanisms.

Until now, Ti_3_C_2_T_*x*_ is extensively employed in desalination and water treatment, and it is essential to develop a new MXene structure and discover other environmental remediation applications of various MXenes. Moreover, several theoretical studies such as DFT predicted superior characteristics of MXenes in desalination and environmental remediation applications [17, 85–87]. Proper experimentation and development of an efficient system are required to confirm the results of these theoretical studies [88–90]. There is no suspicion that commercial MXene-based product will be introduced in the market soon and MXenes will discover their role in the future direction of desalination technology. Based on the current promising results, it can be securely foreseen that MXenes could be the next-generation materials for water treatment and desalination.

## Conclusion

MXenes and MXene-based nanomaterials have offered tremendous advantages, and they have emerged as ideal entrants for future desalination technology. Despite copious hurdles that need to be addressed, based on the promising results from the current research, a remarkable development in the synthesis techniques and applications of these exceptional nanomaterials is anticipated in the near future. For MXenes to be a forerunner in desalination, further research is vital to overawe the existing hurdles. There is no suspicion that MXenes has assured an era of the next-generation 2D nanomaterials and will have a bright future in water purification and environmental remediation.
